# Critical Periods for Prenatal Alcohol Exposure: Evidence From Animal and Human Studies

**Published:** 1994

**Authors:** Claire Coles

**Affiliations:** Claire Coles, Ph.D., is director of Clinical and Developmental Research at the Human and Behavior Genetics Laboratory of the Department of Psychiatry and is director of Psychological Services at the Marcus Development Center of the Department of Pediatrics, the Emory University School of Medicine, Atlanta, Georgia

## Abstract

Birth defects associated with FAS can vary, depending on when during gestation the fetus is exposed and how long the exposure continues. Although defects such as craniofacial abnormalities have been clearly associated with exposure early in pregnancy, behavioral deficits have not been as closely connected with a critical period of pregnancy.

Although in 1968, Lemoine and colleagues (1968) informed the scientific and medical community of alcohol’s potential as a teratogen (i.e., alcohol is a substance that can affect a developing fetus, resulting in physical and neurological damage), Jones and Smith’s (1973) classic article first employed the term “fetal alcohol syndrome” (FAS). It is evident from this and later reports that maternal alcoholism and heavy drinking during pregnancy are associated with readily observable physical defects, called dysmorphia; reduced growth; and damage to the fetus’ nervous system.^1^ However, the extent and pattern of alcohol exposure necessary to produce any of these negative effects are still under investigation.

Some of the most vexing questions about alcohol’s effects on the fetus that remain concern the *timing* of a mother’s drinking during pregnancy; the *duration* of the fetus’ exposure; and whether, if the fetus is exposed early in gestation, it makes any difference to the outcome if the mother discontinues her drinking for the duration of her pregnancy. These questions concern the fetus’ vulnerability during different critical periods of gestation. This article reviews research findings from studies of humans and animals about the effects of alcohol during critical periods in pregnancy.

## Critical Periods

In the first half of this century, the placenta was considered a natural barrier capable of protecting the developing child from exposure to harmful agents, including disease and toxic chemicals. When researchers discovered, during the 1950’s and 1960’s, that certain diseases, such as rubella, and some environmental agents, such as mercury, were teratogenic (Scialli 1992), concern focused on the first 3 months of pregnancy, the first trimester. This stage was considered the risky period—when exposure might lead to birth defects—whereas exposure during the later part of pregnancy was not considered dangerous.

A possible explanation for this view involves the kinds of defects that generally appear if exposure to a teratogen occurs early in pregnancy. The first trimester is the critical period of organogenesis, when the major organs form. Exposure during this time, and particularly during the first 2 months—the embryonic period—can result in dysmorphia. The effects of teratogenic exposure during the second and third trimesters, which include growth retardation and neurological defects (Scialli 1992), are not as obvious and may not be attributed to exposure unless observers are aware of the potential for their occurrence.

There is another, earlier stage—pre-implantation—before the fertilized egg attaches, which used to be considered a safe period because the developing embryo is not yet connected to the maternal bloodstream and, therefore, does not appear to be receiving the teratogenic substance. In the 1980’s, however, research revealed that these ideas may not be completely accurate (Fabro et al. 1984). Many chemicals and organisms are able to cross the placental barrier. These teratogens also have been shown in some studies to affect the fetus during the earliest part of pregnancy that has been studied.

**Figure 1:**
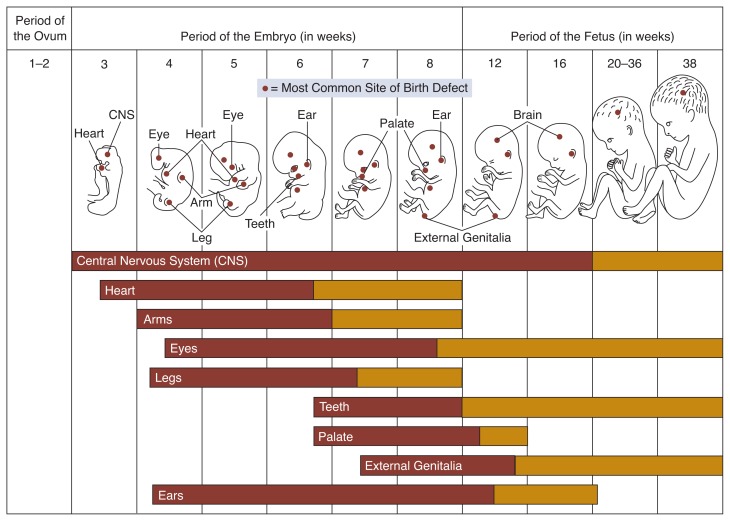
Vulnerability of the fetus to defects during different periods of development. The red portion of the bars represents the most sensitive periods of development, during which teratogenic effects on the sites listed would result in major structural abnormalities in the child. The yellow portion of the bars represents periods of development during which physiological defects and minor structural abnormalities would occur. SOURCE: Adapted from Moore 1993.

Inherent in the idea of a critical period of pregnancy is that the embryo or fetus is vulnerable during specific times when it is undergoing particular developmental processes (figure 1). This period-sensitive vulnerability can be pinpointed when a specific process in development is affected only during these windows of time. For instance, female fetuses may have genital malformations if exposed to androgens (male sex hormones) only around the 10th week of gestation, when the sexual organs are differentiating. Mercury, which has its greatest impact on the developing nervous system, affects the fetus during the second trimester, when nerve cells are proliferating. Other teratogens such as lead or alcohol seem to have negative effects throughout gestation, causing physical anomalies, retarded fetal growth, and neurological damage.

## Animal Studies

Several difficulties arise in attempts to study many of the effects of alcohol exposure on humans during pregnancy. For example, investigators cannot regulate the timing and extent of the subjects’ exposure to alcohol or the strength or purity of the dose, and they cannot control individual differences in response to the alcohol. In contrast, these factors can be controlled in animal studies, where investigators can regulate the dose of alcohol and monitor blood alcohol levels to assure that the exposure is consistent. In addition, to study the timing of effects, animals can be exposed to alcohol throughout gestation, during particular periods of gestation, or only on specific days.

The many social and environmental factors that limit confidence in conclusions drawn from human research also can be eliminated through animal research. For instance, the effects of nutritional deficiencies, which are often associated with heavy alcohol use, can be regulated in animals through feeding a control animal the same diet that the alcoholized animal eats voluntarily. Although this technique may not entirely control the problems of nutritional absorption, it does assure an equal intake of nutrients by both animals.

**Figure 2:**
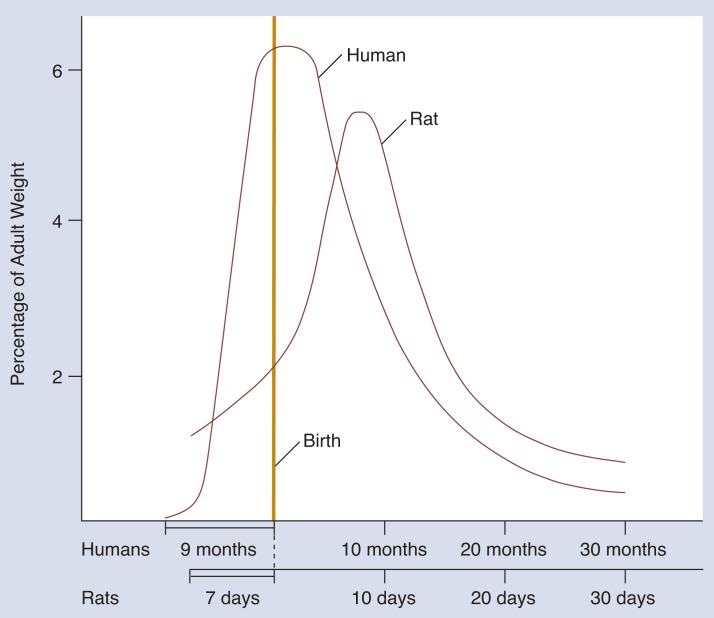
Timing of the brain growth spurt during development in humans and rats. In the rat (scale is measured in days), the brain’s growth peaks *after* birth, whereas in humans (scale is measured in months), the brain’s growth peaks *at* birth. Growth is measured as a percentage of adult weight. As brain growth slows, continued growth in the rest of the body causes the brain’s weight to become a smaller percentage of total adult weight. SOURCE: Modified from Dobbing 1981.

However, there are some problems in using animals as models for the effects of alcohol on humans. First, particular species of animals do not always respond to a substance in the same way humans do, and drugs are often metabolized differently in different species. Second, the gestational process itself may not be completely analogous in different species. For example, when using a rat model to study the behavioral effects of exposure during the brain growth spurt, it is important to realize that this period, which occurs in the human third trimester, occurs after birth in the rat (figure 2). Therefore, alcohol has to be administered to rats in the period right after birth to produce the same results (West and Goodlett 1990).

Finally, the type of effects being investigated may limit the usefulness of animal models. For instance, researchers are concerned with studying the impact of fetal alcohol exposure on affected children’s social behavior and higher cognitive functioning. However, these processes cannot be modeled satisfactorily in most nonhuman species, which do not show deficits in aspects of behavior such as social judgment, language development, or math skills.

## Insight From Animal Studies

Despite inherent limitations, investigations of timing of alcohol exposure in animal models have led to many important insights into physical anomalies in humans. Sulik and Johnston (1983) have investigated the impact of alcohol exposure in mice and established that exposure around day 7 of gestation produces facial dysmorphia and correlated brain alterations equivalent to that seen in children with FAS. Clarren and colleagues (1988) studied effects in primates by exposing two groups of pigtail macaques to a range of doses of alcohol. One group was exposed throughout pregnancy, and the other was exposed after the fifth week of gestation (a time approximately equivalent to late in the first trimester in humans). This study suggested that facial malformations occurred between gestational days 20 and 32 in this species and that *early* exposure was much more damaging for growth and behavior than exposure later in pregnancy, even if a larger dose was given at a later time. The researchers also saw effects on behavior even in the absence of obvious physical defects.

From animal studies, it is clear that early exposure (first trimester) produces significant dysmorphia and neurological damage; however, there also are effects on the nervous system as a result of later exposure only. Using a rat model, Miller (1992) has investigated the effects of exposure during the second half of gestation (equivalent to the second trimester in humans). He notes that brain weight reduction is a consistent finding in animals alcoholized both in this period and in the early postnatal period (equivalent to the third trimester in humans). During the second half of gestation, nerve cells in the neocortex are generated and migrate to the appropriate brain regions. Alcohol exposure appears to affect the timing and pattern of nerve cell generation, both delaying the process and altering the number of cells that are produced. In addition, cell migration patterns are altered so that unusual cell formations can be observed in many areas in the brain, including the hippocampus,^2^ cerebellum, sensory nucleus, and neocortex (Miller 1992).

Third trimester (equivalent) effects have been studied extensively in a rat model by West and Goodlett (1990). This period is of interest because it includes the brain growth spurt, a time of very rapid brain development that occurs in part during the third trimester in humans and postnatally in rats (figure 2). Exposure to alcohol during this period leads to reductions in brain weight and head circumference, presumably associated with alterations in brain structure and function. The number of cells in certain regions of the hippocampus and the cerebellum are reduced. The hippocampus is known to affect learning and memory and the cerebellum to affect motor ability. That these anatomical changes are related to abnormal behavior later in life is suggested by findings that hyperactivity and learning deficits can be observed in physically normal animals exposed to alcohol during this period (West and Goodlett 1990).

## Human Studies

Although dysmorphia, growth, and certain kinds of behavior can be studied in animal models, it is only through human clinical and epidemiologic studies that other important behaviors such as language development can be investigated. However, researchers have to be aware of the limitations of these studies, such as those that arise from the complex pattern and duration of alcohol use usually seen in pregnancy, which is discussed below. To investigate how timing affects outcome and to use statistical techniques most effectively, it would be best if information was available about alcohol exposure at different points during gestation; some fetuses would be exposed in the first trimester, others in the second, and still others in the third. It would be helpful also if levels or doses of drinking were the same so that *only* the timing of exposure was different rather than timing and amount both.

However, women who drink during pregnancy are not motivated by scientific rigor. Instead of adopting drinking patterns that range across trimesters, they are more likely to drink heavily around the time of conception, before realizing that they are pregnant, and to reduce or stop drinking later in pregnancy. Some women reduce drinking because they are aware of the potential problems; others reduce or eliminate alcohol use because of an aversion to its taste or because of the nausea that they feel at this time. Virtually no women begin drinking heavily only during the second or third trimesters or both. Thus, it is impossible to replicate animal results in human clinical studies. Because of these patterns of use and the likelihood that the same women will drink heavily and throughout pregnancy, there are technical difficulties in using statistics to control for *all* of the effects associated with trimester of exposure.

Another difficulty involves the administration of alcohol. Because biological measures of alcohol use such as blood and urine tests are less accurate during pregnancy due to the presence of hormones of pregnancy, it is necessary to rely on a woman’s self-report for the amount and frequency of her drinking. Even when a woman is reporting her drinking as accurately as she can, such reports may be in error, particularly when they involve recall over long periods of time. Because estimates of exposure, particularly exposure during specific periods of gestation, are tentative, human research has focused not on specific days or weeks but on four broad time periods—periconception, first trimester, second trimester, and third trimester—and has asked women to estimate, usually retrospectively, their drinking during these periods. To get a feel for how accurate this information is likely to be, readers are encouraged to try to remember exactly how much they drank during a particular week 6 months ago. Typically, only abstainers and alcoholics are able to do this with any accuracy.

In contrast to most investigators, who ask women about their alcohol use only on one occasion (usually at the time they are recruited into the study), a few investigators have assessed drinking repeatedly and systematically. Day and colleagues (1989) identified women who used alcohol early in pregnancy and repeatedly interviewed them, asking about use during each trimester as well as after the birth of the baby. This method allows relatively accurate estimates of exposure by trimester and allows for the investigation of trimester effects.

Another way to examine the issue of timing of exposure is to take advantage of an experiment that results spontaneously when pregnant women are recruited into programs designed to help them stop drinking. Coles and colleagues (1985) and Larsson and colleagues (1985) have compared physical and behavioral differences in offspring of those women who stopped drinking as a result of intervention, with the offspring of those who did not stop drinking in the second trimester, to estimate the benefits of third trimester abstinence. However, it is necessary to interpret results of such studies cautiously, because it is likely that women who continue to drink despite educational and therapeutic interventions also differ in other ways from women who are able to stop. For instance, those women who continue to drink also are more likely to smoke cigarettes and might be more physically addicted to alcohol than those who were able to stop. Thus, third trimester effects in the offspring of these women could stem from these differences and not only from consumption of alcohol (for more information, see Smith et al. 1986).

## Timing of Alcohol Effects in Humans

### Nonviability

It is probable that heavy, extremely early alcohol exposure often leads to nonviability of the fetus and spontaneous abortion. This is difficult to measure, however, because pregnancy may not have been identified and pregnancy loss may not be recognized.

### Physical Anomalies (Birth Defects)

Because facial dysmorphia occurs during the embryonic period (the first 8 weeks of the first trimester), craniofacial anomalies in human subjects are probably associated with drinking during this initial stage of pregnancy. Clinical support for this period of vulnerability can be inferred from studies that examine the effects of stopping drinking during the second trimester (Coles et al. 1985), in which equivalent physical dysmorphia scores were noted in two separate groups. The first group included children whose mothers drank throughout gestation. The second group included children whose mothers stopped drinking in the second and third trimesters but drank amounts during the first trimester that were equivalent to those consumed during the first trimester by the mothers of the first group (mean amount reported for both groups was 24 drinks a week, with a range of 2 to 150 drinks a week).

In statistical studies of craniofacial anomalies in children exposed to alcohol prenatally, Ernhart and colleagues (1987) report that a relationship between these anomalies and first trimester exposure was evident. The anomalies also were related to later intellectual development in that greater dysmorphia was associated with lower IQ’s. In their longitudinal studies, Graham and colleagues (1988) found minor physical anomalies in alcohol-exposed children, present at birth and also at age 4 years, that were related to heavy drinking in the periconceptual period rather than at midpregnancy. Day and colleagues (1989) found that physical anomalies observed in infants were associated with reports of heavy drinking (defined in their study as at least one drink a day, or about 0.5 ounce of alcohol) in the first 2 months of pregnancy only.

### Effects on Growth

**Figure 3:**
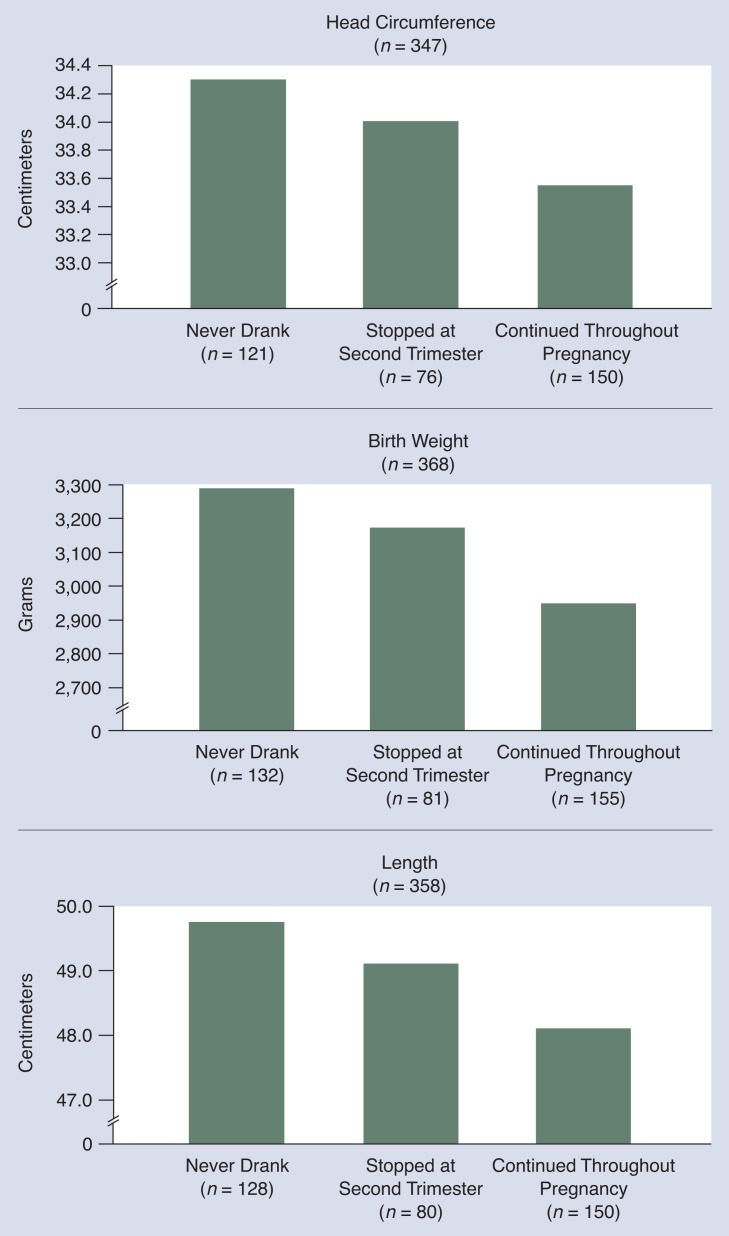
Effects of alcohol exposure on growth. Growth was reduced the most in children whose mothers continued to drink throughout pregnancy. Growth was not as affected in those whose mothers stopped drinking in the second trimester. These data suggest that exposure that continues throughout pregnancy produces fetal growth deficiencies that can be observed at birth. SOURCE: Coles et al. 1991.

Growth is usually measured by birth weight (or weight in older children), head circumference, and length (height). In contrast to the relationship observed with facial dysmorphia, effects on growth appear to be related to exposure later in pregnancy. This relationship is shown in figure 3, which presents data from studies of the effect of discontinuing alcohol and other drug use by the second trimester of pregnancy on neonatal measures of growth. Drugs other than alcohol are mentioned because it cannot be proved that cigarettes and drugs such as marijuana were not used by at least some of the women in the study.^3^

These data suggest that exposure that continues throughout pregnancy produces fetal growth deficiencies that can be observed at birth. When alcohol and other drug use is discontinued by the beginning of the second trimester, children of drinkers may approach the growth of children of nondrinkers (Coles et al. 1985; Rosett et al. 1980). These findings may indicate either that the growth deficit associated with alcohol occurs in the third trimester when the fetus is known to be growing rapidly or that being alcohol-free during this time allows the previously exposed fetus to catch up on growth.

**Figure 4:**
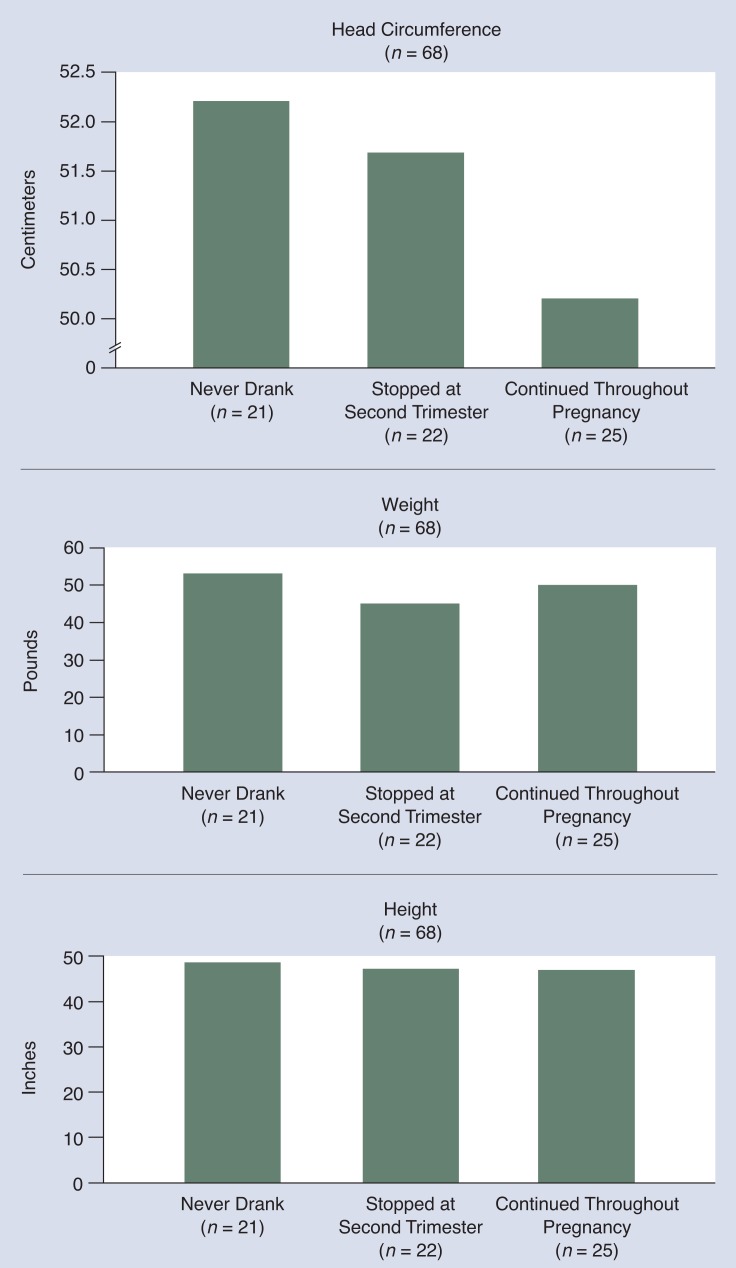
Reevaluation of growth in alcohol-exposed children at 5 to 7 years of age suggests that effects on weight and height are mitigated, whereas effects on head circumference remain. Children whose mothers drank throughout pregnancy show the greatest deficits in head circumference. SOURCE: Coles et al. 1991.

When alcohol-exposed children are studied over time, the negative effects of alcohol exposure on some aspects of growth seem to be mitigated, whereas other aspects of growth are still affected. Figure 4 shows the weight, height, and head circumferences of children in a longitudinal study, some of whom had a diagnosis of FAS or FAE (fetal alcohol effects), and who were reevaluated at early school age (5 to 7 years old). Notice that at this stage, although weight and height are no longer significantly lower in the alcohol-exposed groups, head circumference remains smaller among children who were exposed throughout pregnancy. These data are consistent with the results of animal studies, which found deficits in head circumference in animals exposed during the brain growth spurt (West and Goodlett 1990).

Following a larger sample (650 women) than that shown in figure 4, Day and colleagues (1991) also found that as children aged, there were observable differences in growth rate that appeared to link exposure to alcohol during the second and third trimesters with the effect on height and weight as well as on head circumference. In addition, due to differences in methodology from those used in the study represented in figure 4, these investigators have identified an adverse effect on growth due to first trimester alcohol exposure, and, based on these data, they suggest that there may be more than one mechanism for alcohol’s effects on growth.

### Behavioral Effects

These effects have not been examined as closely in relation to critical periods of exposure as have facial features and growth, primarily because it is much more difficult to make such a connection. Studies of facial dysmorphia have produced a clear connection with alcohol exposure during the first trimester. However, because the relationship between specific brain sites and functions and most human and animal behaviors have not been identified (or may not exist), the same behavior may have more than one “cause.” For instance, a child’s poor attention may result from prenatal or traumatic brain damage, from environmental factors, or from anxiety. Therefore, observation of attention problems in alcohol-exposed children does not point to a particular period of exposure in the same way that facial dysmorphia does.

In addition, children are exposed to different amounts of alcohol at different times during their gestation, so there is a wide range of possible outcomes resulting from fetal alcohol exposure. For example, the severe central nervous system damage associated with reduced head size, or microcephaly, in some FAS children probably results from different maternal drinking patterns from those that cause the mild effects, such as lower IQ scores and alterations in behavior, found in otherwise normal children.

Despite these problems in drawing conclusions from observed behavioral effects, it is possible to tease out some relationships between particular behavioral outcomes and alcohol exposure during different periods of pregnancy. Early, heavy exposure leads to the most severe outcomes and is associated with mental retardation, sensory deficits, and motor problems. More subtle behavioral effects, such as learning disabilities and attention problems, can result from less extensive exposure. For instance, in a prospective sample (mean alcohol use by mothers before pregnancy recognition was approximately 1 drink per day, with a range of 0 to 50 drinks), Streissguth and colleagues (1989) found that reported moderate drinking (mean stated above) either before pregnancy recognition or at midpregnancy was associated with relatively mild later deficits on neuropsychological tests (children scored an average of four points lower than normal on IQ tests). Early, heavier drinking (the upper limit of the range stated above) was found to result in more serious outcomes on the neuropsychological tests even in children without physical effects. In seeming contrast, Larssen and colleagues (1985) found that preschool children who were exposed to alcohol throughout gestation (with a range of two to nine drinks per day) were more likely to show hyperactivity, language problems, and motor deficits in comparison with those whose mothers stopped drinking by the second trimester, which implies that these effects result from later exposure.

**Figure 5:**
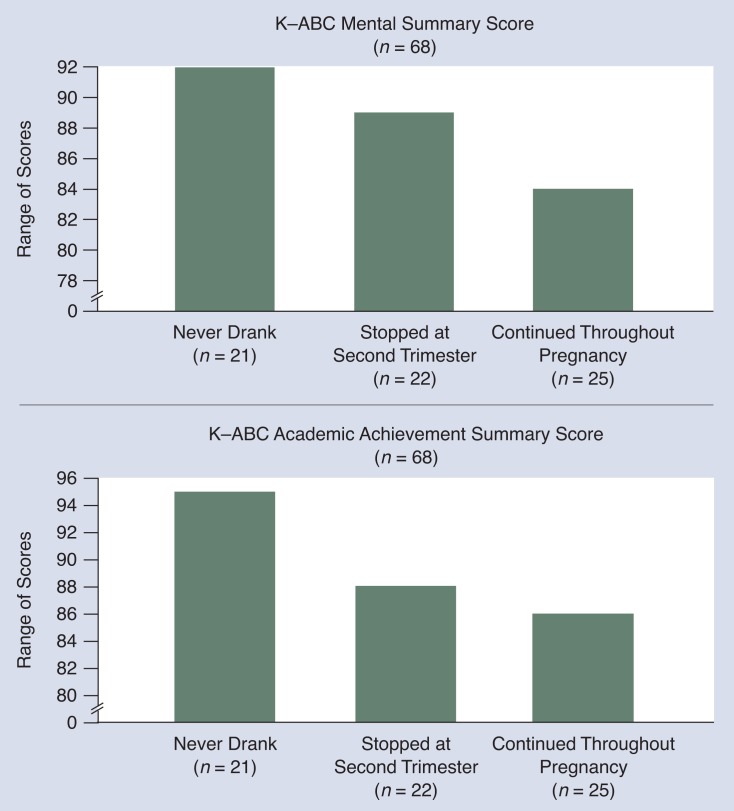
Effects on aptitude and academic achievement in alcohol-exposed children. The Kaufman Assessment Battery for Children (K–ABC) tests IQ and academic achievement for children between 2 and 12 years of age. Children whose mothers stopped drinking during the second trimester of pregnancy are less affected (i.e., have higher IQ and achievement scores) than those whose mothers drank throughout pregnancy. SOURCE: Coles et al. 1991.

Similar results to Larssen and colleagues’ were obtained in a followup of school age children (Coles et al. 1991) that focused on cognition, attention, and behavior (figure 5). In these data, alcohol exposure during any part of pregnancy appears to be associated with poorer academic achievement. Exposure during the third trimester in particular appears to be associated with lower aptitude scores, although the majority of these children cannot be classified as mentally retarded. Effects on aptitude that are associated with third trimester exposure are probably the cumulative effect of alcohol exposure throughout pregnancy. Some of the other deficits that were seen in these children exposed through the third trimester (e.g., poorer attention and sequencing and motor problems) are consistent with those seen in people with damage, such as trauma, to the hippocampus and the cerebellum (Mirsky 1987). These are the brain structures that West and Goodlett (1990) have identified as being affected by third trimester equivalent alcohol exposure in rats. Of course, because the human data results are from correlational studies, it is possible that the observed deficits stem from other factors (e.g., the child-rearing environment) rather than any specific neurological deficits that result from alcohol exposure.

## Conclusions

Despite the extensive research on effects of prenatal alcohol exposure, information about timing of exposure as it relates to the fetus’ vulnerability to particular effects on behaviors remains limited. Due to the nature of the developmental process, there are differences in the amount of knowledge available about specific effects of all kinds. Animal studies and epidemiologic studies strongly suggest that the facial malformation characteristic of FAS results from exposure during the first trimester and, more specifically, during the first 2 months of gestation.

However, the relationship becomes less clear in the examination of growth retardation. It appears that both early exposure (during the first 2 months of pregnancy) and exposure during the third trimester affect growth. Specifically, effects on head circumference—and, by extension, brain growth—appear to be the most consistent and permanent outcomes of exposure during these two periods.

It is much more difficult to examine behavioral effects than physical anomalies, using a trimester approach, due to the nature of behavior development. Correlational studies suggest that early exposure may be more damaging to behavior than is later exposure, but some animal studies indicate that exposure in the third trimester may specifically affect the hippocampus and the cerebellum, leading to deficits in learning and motor skills. However, the many social and physical factors associated with heavy alcohol use by pregnant women as well as the difficulty in studying specific behavioral effects in children limit the interpretations. The available evidence strongly suggests that there may be specific behavioral effects of alcohol exposure during particular periods, but the limitations inherent in both animal and human studies make it difficult to be sure of these outcomes.

It also is clear from these data that although a mother’s heavy alcohol use in pregnancy is potentially damaging to the fetus, her stopping use is likely to have a beneficial outcome even on many of the functions (e.g., growth and behavior) that were affected by earlier drinking. In the future, animal studies may illuminate specific brain structures and patterns of behavior that are impacted by certain patterns of alcohol exposure (West and Goodlett 1990). It may be possible to relate such findings to similar brain areas and behavior patterns in humans and thereby greatly increase understanding of these problems.

## Footnotes

1 The terms “heavy drinking” and “alcoholism” do not refer to specific amounts of drinking or conditions. The amount of alcohol that must be consumed by a pregnant woman in order to produce effects in her fetus has never been determined.

2 For a definition of this term and other terms used in this article, see the glossary on page 25. Also, to locate specific areas of the brain mentioned in this article, see the diagram of the brain on page 50.

3 The effects of cigarettes and drug use cannot be ruled out as factors in the data presented in figures 3, 4, and 5.

## Glossary

Cerebellum: The second largest portion of the brain. It is involved in motor functions such as maintenance of posture, coordination, and balance and also may be connected with emotional development.
Correlational studies: Studies of humans that relate one behavior or interaction with the environment to observed physiological or psychological effects. Because no study of humans can eliminate other possible causes of such effects (something that can be done in controlled animal studies), only correlations can be drawn between a behavior or teratogen and an observed effect.
Hippocampus: A component of the limbic system within the brain that is involved in emotional behaviors related to survival, such as flight or fight responses. The hippocampus is associated with memory, particularly with the learning of new information and of sequentially presented information.
Neocortex: The most recently evolved portion of the cerebral cortex. The cerebral cortex (including the neo-cortex) covers the surface of the cerebrum (the largest portion of the brain) and is responsible for higher mental functions, general movement, perception, behavioral reactions, and the integration of these functions.
Sensory nucleus: The nucleus of termination of the sensory fibers of a peripheral nerve. The peripheral nerves carry information from the sense organs (eyes, ears, skin) to the brain. These neurons terminate in clusters of cells called nuclei. Information is then transferred from these sensory nuclei to other brain structures.

## References

[b1-arhw-18-1-22] Clarren SK, Astley SJ, Bowden DM (1988). Physical anomalies and developmental delays in non-human primates exposed to weekly doses of ethanol during gestation. Teratology.

[b2-arhw-18-1-22] Coles CD, Smith IE, Fernhoff PM, Falek A (1985). Neonatal neurobehavioral characteristics as correlates of maternal alcohol use during gestation. Alcoholism: Clinical and Experimental Research.

[b3-arhw-18-1-22] Coles CD, Brown RT, Smith IE, Platzman KA, Erickson S, Falek A (1991). Effects of prenatal alcohol exposure at school age: I. Physical and cognitive development. Neurotoxicology and Teratology.

[b4-arhw-18-1-22] Day NL, Jasperse D, Richardson G, Robles N, Sambamoorthi U, Scher M, Staffer D, Cornelius M (1989). Prenatal exposure to alcohol: Effect on infant growth and morphological characteristics. Pediatrics.

[b5-arhw-18-1-22] Day NL, Robles N, Richardson G, Geva D, Taylor P, Scher M, Staffer D, Cornelius M, Goldschmidt L (1991). The effects of prenatal alcohol use on the growth of children at three years of age. Alcoholism: Clinical and Experimental Research.

[b6-arhw-18-1-22] Dobbing J, Davis JA, Dobbing J (1981). The later development of the brain and its vulnerability. Scientific Foundations of Paediatrics.

[b7-arhw-18-1-22] Ernhart CB, Sokol RJ, Martier S, Moron P, Nadler D, Ager JW, Wolf A (1987). Alcohol teratogenicity in the human: A detailed assessment of specificity, critical period, and threshold. American Journal of Obstetrics and Gynecology.

[b8-arhw-18-1-22] Fabro S, McLachlan JA, Dames NM (1984). Chemical exposure of embryos during the preimplantation stages of pregnancy: Mortality rate and intrauterine development. American Journal of Obstetrics and Gynecology.

[b9-arhw-18-1-22] Graham JM, Hanson JW, Darby BL, Barr HM, Streissguth AP (1988). Independent dysmorphology evaluations at birth and 4 years of age for children exposed to varying amounts of alcohol in utero. Pediatrics.

[b10-arhw-18-1-22] Jones KL, Smith DW (1973). Recognition of fetal alcohol syndrome in early infancy. Lancet.

[b11-arhw-18-1-22] Larsson G, Bohlin A–B, Tunell R (1985). Prospective study of children exposed to variable amounts of alcohol in utero. Archives of the Diseases of Children.

[b12-arhw-18-1-22] Lemoine P, Harousseau H, Borleyru JP, Menuet JC (1968). Les enfants de parents alcooliques: Anomalies observees a propos de 127 cas. Ouest Medical.

[b13-arhw-18-1-22] Miller MW, Miller M (1992). Effects of prenatal exposure to ethanol on cell proliferation and neuronal migration. Development of the Central Nervous System: Effects of Alcohol and Opiates.

[b14-arhw-18-1-22] Mirsky AF (1987). Behavioral and psychophysiological markers of disordered attention. Environmental Health Perspective.

[b15-arhw-18-1-22] Moore KL, Berger KS (1980). The Developing Human [1993]© Philadelphia . W.B. Saunders Co. The Developing Person.

[b16-arhw-18-1-22] Rosett HL, Weiner L, Zuckerman B, McKinlay S, Edelin KC (1980). Reduction of alcohol consumption during pregnancy with benefits to the newborn. Alcoholism: Clinical and Experimental Research.

[b17-arhw-18-1-22] Scialli AR (1992). A Clinical Guide to Reproductive and Developmental Toxicology.

[b18-arhw-18-1-22] Smith IE, Lancaster JS, Moss-Wells S, Coles CD, Falek A (1986). Identifying high risk pregnant drinkers: Biological and behavioral correlates of continuous heavy drinking during pregnancy. Journal of Studies on Alcohol.

[b19-arhw-18-1-22] Streissguth AP, Bookstein FL, Sampson PD, Barr HM (1989). Neurobehavioral effects of prenatal alcohol: Part III. PLS analyses of neuropsychologic tests. Neurotoxicology and Teratology.

[b20-arhw-18-1-22] Sulik KK, Johnston MC (1983). Sequence of developmental alterations following acute ethanol exposure in mice: Craniofacial features of the fetal alcohol syndrome. American Journal of Anatomy.

[b21-arhw-18-1-22] West JR, Goodlett CR (1990). Teratogenic effects of alcohol on brain development. Annals of Medicine.

